# Immigration Status, Educational Level, and Perceived Discrimination in Europe

**DOI:** 10.3390/ijerph20032222

**Published:** 2023-01-26

**Authors:** Hafifa Siddiq, Najmeh Maharlouei, Babak Najand, Arash Rahmani, Hossein Zare

**Affiliations:** 1 School of Nursing, Charles R. Drew University of Medicine and Science, Los Angeles, CA 90059, USA; 2 Marginalization-Related Diminished Returns, Los Angeles, CA 90059, USA; 3 Department of Health Policy and Management, Johns Hopkins Bloomberg School of Public Health, Baltimore, MD 21205, USA; 4 School of Business, University of Maryland Global Campus (UMGC), Adelphi, MD 20783, USA

**Keywords:** discrimination, educational level, immigration, Europe

## Abstract

Background: Multiple studies have been conducted to test the moderating effect of immigration on the positive health results yielded through educational attainment. However, no study has been conducted to examine the role of immigration as a moderator in the association between educational level and perceived discrimination in Europe. Aim: We aimed to study whether an inverse association exists between educational level and perceived discrimination in European countries and whether immigration status moderates the association between educational level and perceived discrimination. Methods: Data from the 10th round of the cross-sectional European Social Survey (ESS) were used in this cross-sectional study. A total of 17,596 participants between 15–90 years old who lived in European countries were included. The independent variable was educational level, a categorical variable, and the dependent variable was perceived discrimination. Immigration status was the moderator, and age and sex were confounders. Results: Of 17,596 participants, 16,632 (94.5%) were native-born and 964 were immigrants (5.5%). We found that higher levels of educational level were protective against perceived discrimination, which was also found in immigrant participants; however, the effect was weaker. Conclusions: This study found that educational level was a protective factor against perceived discrimination. This effect, however, was more robust in the native-born participants than in their immigrant counterparts.

## 1. Introduction

Socioeconomic status (SES) indicators are essential factors that the population and individuals’ health. Several studies have shown that people with higher SES have lower chronic diseases, better mental health, and longer life expectancy [1,2]; however, this effect is mediated by perceived discrimination [3]. However, the effect of SES on health outcomes has been shown to be mediated by psychological distress [4], social support [5], and perceived discrimination [3]. Marginalized populations, including immigrants, experience healthcare inequities and health disparities when compared to their native-born counterparts. In this study, we focused on whether immigration status moderates the effect of educational level on perceived discrimination.

Various theoretical definitions have been proposed for discrimination in sociological literature. Oxford Sociology Bibliographies describes discrimination as an “action or practice that excludes, disadvantages, or merely differentiates between individuals or groups of individuals on the basis of some ascribed or perceived trait” [6]. Furthermore, in the Oxford Dictionary of Sociology, Scott suggests that discrimination can be broadly considered ‘treating unfairly’ [7]. Early sociologists, such as William G. Sumner and Franklin H. Giddings, have viewed discrimination as an expression of ethnocentrism, which defines discrimination as a cultural phenomenon of ‘dislike of the unlike’. This interpretation is different from more recent views that defines discrimination as a pattern of dominance and oppression, viewed as expressions of a struggle for power and privilege [7]. More relevant to our study, Krieger 2014 [8] defined discrimination as follows: “the process by which a member, or members, of a socially defined group is, or are, treated differently (especially unfairly) because of his/her/their membership of that group” [9], extended this definition with notions from the Concise Oxford Dictionary of Sociology that discrimination involves not only “socially derived beliefs each [group] holds about the other” but also “patterns of dominance and oppression, viewed as expressions of a struggle for power and privilege” [10]. Finally, Krieger concludes that discrimination is “a socially structured and sanctioned phenomenon, justified by ideology and expressed in interactions between individuals and institutions, that preserves privileges for dominant groups at the cost of deprivation for others” [8]. We adopted this last statement as our working definition in our study. The effect of perceived discrimination on health has been documented in a longitudinal study conducted by Fuller-Rowell et al., which showed that about 14% of the longitudinal association between SES and self-rated health was explained by perceived discrimination [3].

Perceived discrimination may be related to intersecting identities around race/ethnicity, indigenous status, age, gender, sexuality, disability, and immigration status [8,11,12,13,14,15,16]. A study based on the data of 4733 White American citizens demonstrated that higher educational level was positively associated with higher perceived discrimination [17]. Another study by Zhang et al. found higher levels of perceived discrimination in Asian-American populations with a college degree or above than those with lower educational levels [18]. 

Educational level promotes the health status of people. Grosse and Auffrey, in 1989, concluded that literacy is a crucial tool for adapting to different conditions [19]. This effect, however, is tenuous among people who belong to minority groups [20,21], a phenomenon called marginalization-related diminished returns (MDRs) [11,12]. In a study of 25,659 US adults, Assari et al. demonstrated that people with high educational level had a protective effect against developing cardiac disease; however, this effect was weaker in Hispanic and African American individuals than non-Hispanic White people [22]. Multiple studies replicated MDRs, defined as the attenuated positive impact of educational level on the health status of marginalized groups in various settings [23,24,25,26,27,28,29,30,31,32,33,34]. Hence, people from marginalized groups have limited access to education due to structural racism and racial discrimination. They may take less advantage of educational attainment partly because they feel discriminated against [3,8,35,36]. The differential return of higher levels of education is different from lower access to education. Historically, most researchers have argued that minorities have fewer opportunities to attain advanced education due to systematic racism/discrimination, particularly for lower-income individuals who work and have more difficulty investing time into higher education.

Previous research demonstrated that immigrants experience high levels of discrimination in Europe [37,38,39], which even extends to their next generations [40]. Immigrants in Europe face economic and social challenges [41,42] that can affect their health and economic outcomes [43]. Research in the field of Marginalized-related Diminished Returns (MDRs) previously showed that immigration can be considered a moderator for the association of SES resources and health and economic outcomes in the US [44,45,46,47]. Within the European context, immigration was shown to moderate the association between education and self-rated health [48]. These diminished returns have been attributed to structural barriers that prevent marginalized minority groups from gaining equal benefits from their SES resources [49,50]. Therefore, in this study, we aimed to assess whether and to what extent immigration can modulate the protective effect of educational level on perceived discrimination in European countries.

Multiple studies have been conducted to test the moderating effects of immigration on positive health results from educational level [30,51,52,53,54]. To the best of our knowledge, no study has been identified that examines the role of immigration as a moderator on the association between educational level and perceived discrimination in Europe. Although we know that the association between educational level and perceived discrimination exists [17,18,55,56], no previous European studies have examined the role of immigration as a moderator in the association between educational level and perceived discrimination. Related research was conducted by Bakhtiary, who used data from 30 European countries and suggested that SES affects health status. However, this effect depends on immigration status. Based on this study, future research is needed to evaluate the moderating role of immigration on the phenomenon of MDRs [57].

To address this gap in the literature, we aimed to study whether an inverse association exists between educational level and perceived discrimination in European countries and if immigration status moderates the association between educational level and perceived discrimination. First, we hypothesized an inverse association between educational level and perceived discrimination. Second, we hypothesized that this association would be weaker for immigrants.

## 2. Methods

This study used data from the 10th round of the cross-sectional European Social Survey (ESS) [57], conducted from September 2020 to the end of January 2022 in ten European countries, including Bulgaria, Czechia, Estonia, Finland, France, Croatia, Hungary, Lithuania, Slovenia, and Slovakia.

ESS is a cross-national survey founded in 2001 to study whether the MDRs pattern applies to other countries with different contexts from the US. Unlike previous rounds, in which data were gathered through an hour-long face-to-face interview, a self-administered questionnaire was used in the 10th round due to the COVID-19 pandemic. Additionally, some countries used video interviews as a backup plan [57]. Countries that did not report data on the participants’ gender, educational level, perceived discrimination, and immigration status were excluded from the analysis. 

### 2.1. Study Population

The total sample was 17,596 individuals aged 15–90 who lived in one of the countries included in the 10th round of this survey, either native residents (16,632; 94.5%) or immigrant residents (964; 5.5%).

### 2.2. Measures

Evaluating the association between educational level and perceived discrimination, we selected the years of formal education as an independent and the latter as the outcome variables. Although there are differences between educational systems of various countries participated in this study, this issue was addressed in the design of this study by harmonizing the educational levels between different educational systems using International Standard Classification of Education (ISCED). Hence, the educational level in our study is a universal variable that applies to all these countries included. Other variables, including age, gender, and country of residency, are considered confounding factors. Immigration status was the moderator.

**Dependent Variable:** The outcome was measured by asking a question from the respondents about discrimination. The question was, “*Do you describe yourself as a member of a group that is discriminated against in this country?”*. Here, “yes” and “No” answers were recorded as “1” and “0”, respectively.

**Independent Variable:** Educational level: The International Standard Classification of Education (ISCED) was used to classify the participants’ educational levels. ISCED has nine categories, coding from “1” to “9”. Code “1” was used for participants who did not complete primary education, “2” for those who completed primary education or less than two years of vocational studies, “3” for lower secondary education, “4” for lower-tier upper secondary education, “5” for upper-tier upper secondary education, “6” for advanced vocational sub-degrees, “7” for those with bachelor or equivalent degrees, “8” for those who had a master degree or equivalent, and “9” for those had a doctoral or equivalent degree. 

**Moderator:** Immigration status was the moderator variable determined through the following question. “*Were you born in [country]?*”. “No” answers, equivalent to Immigrant status, were recorded as 1, and “Yes” answers, equal to native-born participants were coded as 0.

**Confounders:** In this study, age and sex were considered confounders. Age (years) was a continuous variable ranging from 15 to 90, and sex was a dichotomous variable, coded 1 for men and 0 for women. Other potential confounders, such as income or behavioral traits, can be considered for this study, which we decided not to include because of lacking data or risk of overadjustment [58,59,60].

### 2.3. Data Analysis

Statistical Package for Social Sciences (SPSS) version 23 (IBM Inc., NY, USA) was used for data analysis. Descriptive statistics were reported as frequency (%) and mean (± standard deviation) for ordinal and numerical variables. The logistic regression models reported adjusted unstandardized regression coefficients (b) and corresponding 95% confidence intervals (CIs), as well as *p* values. Four multivariable logistic regression models with perceived discrimination as the primary outcome were used. *Model 1* and *Model 2* were performed in the pooled sample. *Model 1* did not include any interaction term; however, *Model 2* included immigration status by educational level as an interaction term. *Model 3* and *Model 4* were specified in the native-born participants and immigrants, respectively. We tested our central hypothesis in *Model 2* with the significance of the interaction term between immigration status and educational level. This approach tests population differences in correlates of factors such as educational level or perceived discrimination [11,35,61,62,63,64,65,66,67,68,69,70,71].

We applied a statistical method, which is a standard procedure to test diminished return and has been widely applied in previous research [72,73,74]. We tested the main effects of educational level and immigration on perceived discrimination in model 1. It is necessary to test the main effect in a separate model (*model 1*) before testing interactions in model 2. One cannot test statistical interactions without priory testing the main effect [75]. Because life conditions are different for various groups and the levels of exposure and vulnerability of groups toward risk and resilient factors can be different, one need to do the stratified model and include all covariates model 3 and 4. By doing this, a researcher can observe the differential roles of covariates across social groups. Another function of this stratified model is to confirm the finding of interaction observed in model 2 [76,77]. The main effect shows the overall effect in the population, assuming no heterogeneity in the effects across groups [78]. However, in the presence of any hypothesis about innate difference between groups, the main model should be followed or expanded by interaction models. The interaction model is an extension of the previous model, which evaluates the significance of the difference (moderating effect) between groups.

**Robustness check.** A sensitivity analysis was also conducted using model tests with the following modifications. The educational level variable was recoded from 0 to 3, with the following groups. Code ”0” was used for participants in education category “1”, who did not complete primary education, as well as category “2” for those who completed primary education or less than two years of vocational studies, and education category “3” for lower secondary education. Code 1 was used for upper secondary education, comprising education category “4” for lower-tier upper secondary and category “5” for upper-tier upper secondary education. Code 3 was for advanced education, composed of categories “6” for advanced vocational sub-degrees, category ”7” for those with bachelor’s or equivalent degrees, education category ”8” for those who had a master’s degree or equivalent, and education category ”9” for those had a doctoral or equivalent degree. To analyze age, we ran the models with the following categorical age groups: 15–24, 25–49, 50–65, and 65+. We also tested models with age or age and age square. We also ran models with dummy variables for countries as covariates. As the results did not change for our interaction terms that reflected the MDRs, only one set of results was reported.

## 3. Results

Table 1 shows the demographic characteristics of all participants based on immigration status. The frequency of men and women participating in this study was similar between native-born and immigrant respondents. However, more immigrants reported perceived discrimination compared to their native-born counterparts (13.4% vs. 7.9%). Regarding the country of origin of participants in each country, Bulgaria and Estonia reported the lowest and the highest rate of immigrant participants, i.e., 0.7% and 13.7%, respectively. Table A1 in the Appendix A shows the number of participants from each country.

### 3.1. Logistic Regression in the Overall Sample

Table 2 summarizes the results of two logistic regressions in the pooled sample, with perceived discrimination as the primary outcome. *Model 1*, which did not include any interaction term, shows the main effect of educational level; in contrast, *Model 2* considered the interaction between immigration status and educational level. 

According to the information revealed by *Model 1*, the pooled sample, a higher educational level was correlated with lower odds of perceived discrimination. *Model 2* demonstrates a statistically significant interaction between educational level and immigration status on the reported perceived discrimination. Therefore, the protective effect of educational level against perceived discrimination is more robust in native-born participants than in their immigrant counterparts.

### 3.2. Stratified Logistic Regressions 

Table 3 shows the logistic regression results in native-born participants (*Model 3*) and the logistic regression in immigrants (*Model 4*). In these models, educational level was the independent variable, and perceived discrimination was the outcome (dependent variable). *Model 3* shows that in native-born participants, the higher the educational level they reported, the lower the discrimination they perceived. However, *Model 4* showed that educational level did not protect immigrant respondents against perceived discrimination.

## 4. Discussion 

This analysis included data from 17,596 European residents aged 15–90 who participated in the 10th round of the cross-sectional European Social Survey (ESS), and participants were predominantly native-born residents (16,632; 94.5%). Results revealed that as the educational level rises, perceived discrimination falls. Interestingly, our findings are inconsistent with the results of studies by Das, who reported that white US citizens with a higher educational level faced more discrimination [17]. Some other studies suggest that perceived discrimination generally increases with higher educational level among ethnic minorities in the U.S. For example, Zhang et al. found that the Asian-American population with a college degree or above reported a higher level of perceived discrimination than those with a lower educational level [18]. Higher educational level among immigrant groups in the US may be associated with an increased level of acculturation. In another US-based study examining the relationship between perceived discrimination and mental illness, higher levels of acculturation led to a significant increase in discrimination’s association with mental illness [79].

We found that immigrants also benefited from the protective effect of educational level against discrimination; however, the effect is less robust than the native-born population. A study conducted on immigrants in the US showed that, similar to other marginalized groups, the protective effects of higher educational levels is less remarkable in decreasing the risk of psychological distress and chronic diseases and improving subjective health status for immigrant populations [51]. A study by Steinmann on Turkish and Polish immigrants residing in Germany showed that the frequency of reporting discrimination among participants with higher educational levels depended on the “bright boundaries” [80] they faced; the brighter the boundaries they felt, the harder they found it to assimilate. For instance, Turkish immigrants reported discrimination more frequently than their Polish immigrant counterparts because Turkish immigrants thought of themselves as a different community [81]. On the other hand, a study conducted among undergraduate students of Ethiopian origin in Israel showed that perceived discrimination motivated students of Ethiopian origin to pursue higher education. Thus, the association observed in cross-sectional studies may have unexpected consequences longitudinally [82].

There is extensive evidence of discrimination against immigrants in European countries [83]. Borgonovi and Pokropek, using data from four rounds of the European Social Survey conducted in several European countries between 2010 and 2016, indicated that individuals with a higher education level are associated with lower levels of opposition to migrants [84]. Noticeably, feelings of threat mediated around 60% of the effect of the educational level on opposition to migrants. Additionally, a larger foreign-born population was significantly associated with a steeper education gradient for feeling threatened. Such attitudes do not necessarily equal greater opposition to migration, because feelings of threat are less associated with support for restrictive migration measures in countries with high numbers of foreign-born residents [84]. In studies of discrimination in European countries, it is important to identify relevant populations of immigrants, as various ethnic-racial groups, such as European immigrants and visible or non-European immigrants, do not experience it to the same level [83]. Heath and Cheung showed that non-European minorities experience ethnic penalties in accessing the job market. These disadvantages extend beyond the next generations despite the educational progress of children of immigrants [85]. Nuances such as phenotypic features, including skin color [86,87], religion [88,89,90], and accent [91,92], may have a role in the differential treatment of European and non-European immigrants and their subsequent perceived discrimination. Unfortunately, information about the characteristics of immigrants, such as region or country of origin, was not available in our dataset.

Immigration status should not be considered a confounder or covariate but a complex contextual variable. Immigration status alone may be a proxy of diverse personal experiences, including an individual’s cultural background and perceived marginalization [51]. However, immigration status as a variable may not be enough to capture more nuanced factors, including length of stay, citizenship status, generation status, or even context of migration (forced displacement, including asylum or refugee status). Additionally, complex associations may be observed for perceived discrimination across sub-groups of immigrants. For example, skin color and accent may explain why some groups of immigrants experience discrimination. Halanych et al. analyzed data from 1800 African American (45%) and White participants (55%) and showed a direct positive association between educational level and perceived discrimination [56]. While these associations were more prevalent among African American participants, sub-group differences such as gender, age, and income levels exist for African American and White Americans. Another study based on data from 2606 European adults found that perceived discrimination is associated with poor health outcomes among first-generation immigrants from low-income countries who live in European countries but not among their descendants [93]. These studies indicate the complexity of experiences of perceived discrimination among immigrant groups and the heterogeneity of perceived discrimination within and across immigrant groups that should be considered. 

While studies have established the association between perceived discrimination and health, there is a need to examine structural discrimination on health outcomes beyond interpersonal discrimination [8]. There is a need to investigate the social and political influences that give rise to perceived discrimination. While restrictive immigration policies have been found to be associated with higher levels of discrimination among immigrants [94], there is a need to examine how neighborhood factors such as ethnic enclaves and sanctuary cities may be protective against perceived discrimination. Moreover, there is a need to incorporate unique dimensions of the immigration experience, including citizenship and generation status. 

## 5. Limitations

This study was cross-sectional. Perceived discrimination may be a driver for pursuing higher educational level [82]. Moreover, the data included no information regarding immigrants’ length of stay. It is shown that new immigrant responders report perceived discrimination more frequently [81]. Additionally, we analyzed data from 10 European countries with different policies regarding immigrants. There is a need to study how much immigrants feel assimilated into the newly adopted culture [81]. In addition, the response rate in this study was slightly below 50%, which may cause sampling bias. Additionally, there may be several potential confounders that were not controlled in this study. Furthermore, no information was available regarding the country where they got their educational degree. According to a US study by Zhang et al., immigrants who had completed their education outside the US reported less discrimination than those who studied in the US [18]. Further studies are needed to evaluate if there are any differences between native-born and immigrants regarding the positive impact of educational level on health outcomes.

## 6. Conclusions

This study found that educational level was a protective factor against perceived discrimination among European adults. Despite the overall protective effect of educational level against discrimination, this effect was more robust in the native-born participants than in their immigrant counterparts. Future studies are needed to evaluate if there are any differences between native-born and immigrants in European countries regarding the positive impact of educational level on other health outcomes. 

## Figures and Tables

**Table 1:** Distribution of demographic factors overall and by immigration status.

		All n = 17,596	Native-Born Participants n = 16,632 (94.5)	Immigrant Participants n = 964 (5.5)
Sex		Frequency (%)	Frequency (%)	Frequency (%)
Age * (Mean, SD)		50.87 (±18.44)	50.68 (±18.49)	54.12 (±17.28)
Sex (%)				
	Women	979 (55.2)	9194 (55.3)	525 (54.5)
	Men	7877 (44.8)	7438 (44.7)	439 (45.5)
Educational level * (%)				
	Level 1	53 (0.3)	40 (0.2)	13 (1.3)
	Level 2	502 (2.9)	451 (2.7)	51 (5.3)
	Level 3	2109 (12.0)	1992 (12.0)	117 (12.1)
	Level 4	8528 (48.5)	8126 (48.9)	402 (41.7)
	Level 5	1060 (6.0)	1001 (6.0)	59 (6.1)
	Level 6	872 (5.0)	829 (5.0)	43 (4.5)
	Level 7	1983 (11.3)	1886 (11.3)	97 (10.1)
	Level 8	2310 (13.1)	2144 (12.9)	166 (17.2)
	Level 9	179 (1.0)	163 (1.0)	16 (1.7)
Perceived discrimination *				
	No	16,296 (92.6)	15,461 (93.0)	835 (86.6)
	Yes	1300 (7.4)	1171 (7.0)	129 (13.4)

Data were reported as frequency (%) or Mean (±SD) as indicated. * *p* < 0.05 for comparison of immigrant and native-born groups. For education, 1 = did not complete primary education, 2 = completed primary education or less than two years of vocational studies, 3 = lower secondary education, 4 = lower tier upper secondary, 5 = upper tier upper secondary, 6 = advanced vocational, sub-degree, 7 = bachelor or equivalent degrees, 8 = master degree or equivalent, and 9 = doctoral or equivalent degree.

**Table 2:** Pooled sample logistic regression models.

	OR		S.E.	95% C.I. for OR	Sig
Model 1 (Main Effects)					
Age	0.989	0.002	0.986	0.992	<0.001
Men	0.918	0.059	0.818	1.030	0.146
Immigrant	1.996	0.101	1.637	2.434	<0.001
Educational level (0 as reference)					<0.001
Level 2	0.341	0.328	0.179	0.648	0.001
Level 3	0.248	0.309	0.135	0.454	<0.001
Level 4	0.134	0.304	0.074	0.243	<0.001
Level 5	0.159	0.323	0.084	0.300	<0.001
Level 6	0.167	0.327	0.088	0.316	<0.001
Level 7	0.135	0.315	0.073	0.249	<0.001
Level 8	0.157	0.311	0.085	0.289	<0.001
Level 9	0.121	0.443	0.051	0.288	<0.001
**Model 2 (Interactions)**					
Men	0.917	0.059	0.817	1.030	0.144
Age	0.989	0.002	0.986	0.992	<0.001
Immigrant	0.543	0.739	0.127	2.311	0.408
Educational level (1 as reference)					<0.001
Level 2	0.276	0.362	0.136	0.561	<0.001
Level 3	0.186	0.343	0.095	0.364	<0.001
Level 4	0.095	0.338	0.049	0.185	<0.001
Level 5	0.115	0.357	0.057	0.231	<0.001
Level 6	0.118	0.361	0.058	0.239	<0.001
Level 7	0.100	0.348	0.050	0.197	<0.001
Level 8	0.104	0.346	0.053	0.205	<0.001
Level 9	0.082	0.493	0.031	0.217	<0.001
Educational level × Immigrant					0.008
Level 2 × Immigrant	1.516	0.870	0.276	8.333	0.632
Level 3 × Immigrant	2.081	0.796	0.437	9.904	0.357
Level 4 × Immigrant	4.324	0.756	0.982	19.034	0.053
Level 5 × Immigrant	3.681	0.851	0.694	19.534	0.126
Level 6 × Immigrant	4.707	0.858	0.875	25.312	0.071
Level 7 × Immigrant	2.524	0.832	0.494	12.889	0.266
Level 8 × Immigrant	6.721	0.770	1.485	30.414	0.013
Level 9 × Immigrant	5.140	1.119	0.573	46.065	0.143

For education, 1 = did not complete primary education, 2 = completed primary education or less than two years of vocational studies, 3 = lower secondary education, 4 = lower tier upper secondary, 5 = upper tier upper secondary, 6 = advanced vocational, sub-degree, 7 = bachelor or equivalent degrees, 8 = master degree or equivalent, and 9 = doctoral or equivalent degree.

**Table 3:** Stratified logistic regression models.

	OR	S.E.	95% C.I. for EXP(B)		Sig.
Model 3 (Native residents)					
Men			0.793	1.011	
Age	0.990	0.002	0.987	0.993	<0.001
Educational level (1 as reference)					<0.001
Level 2	0.277	0.361	0.137	0.563	<0.001
Level 3	0.193	0.342	0.099	0.377	<0.001
Level 4	0.098	0.338	0.051	0.191	<0.001
Level 5	0.118	0.357	0.059	0.238	<0.001
Level 6	0.121	0.361	0.059	0.244	<0.001
Level 7	0.103	0.348	0.052	0.204	<0.001
Level 8	0.107	0.346	0.055	0.212	<0.001
Level 9	0.085	0.493	0.032	0.223	<0.001
Constant	1.093	0.352			0.799
Model 4 (Immigrant)					
Men	1.184	0.194	0.809	1.733	0.385
Age	0.972	0.006	0.961	0.983	<0.001
Educational level (1 as reference)					0.174
Level 2	0.356	0.806	0.073	1.730	0.201
Level 3	0.305	0.739	0.072	1.297	0.108
Level 4	0.329	0.693	0.085	1.278	0.108
Level 5	0.387	0.785	0.083	1.805	0.227
Level 6	0.452	0.796	0.095	2.153	0.319
Level 7	0.190	0.774	0.042	0.866	0.032
Level 8	0.581	0.703	0.146	2.306	0.440
Level 9	0.377	1.018	0.051	2.770	0.337
Constant	1.652	0.767			0.513

For education, 1 = did not complete primary education, 2 = completed primary education or less than two years of vocational studies, 3 = lower secondary education, 4 = lower tier upper secondary, 5 = upper tier upper secondary, 6 = advanced vocational, sub-degree, 7 = bachelor or equivalent degrees, 8 = master degree or equivalent, and 9 = doctoral or equivalent degree.

## Data Availability

Data supporting reported results can be found at https://ess-search.nsd.no/en/study/172ac431-2a06-41df-9dab-c1fd8f3877e7 (accessed on 22 November 2022). All individuals can access the data with no limitation.

## Appendix A

**Table A1:** Distribution of countries overall and by immigration status.

	All n = 17,596	Native Participants n = 16,632 (94.5)	Immigrant Participants n = 964 (5.5)
	Frequency (%)	Frequency (%)	Frequency (%)
Bulgaria	2617 (14.9) (100)	2599 (15.6) (99.3) ^§^	18 (1.9) (0.7)
Czechia	2448 (13.9) (100)	2378 (14.3) (97.1) ^§^	70 (7.3) (2.9)
Estonia	1538 (8.7) (100)	1328 (8.0) (86.3) ^§^	210 (21.8) (13.7)
Finland	1569 (8.9) (100)	1514 (9.1) (96.5) ^§^	55 (5.7) (3.5)
France	1949 (11.1) (100)	1726 (10.4) (88.5) ^§^	223 (23.1) (11.5)
Croatia	1532 (8.7) (100)	1357 (8.2) (88.6) ^§^	175 (18.2) (11.4)
Hungary	1791 (10.2) (100)	1765 (10.6) (98.5) ^§^	26 (2.7) (1.5)
Lithuania	1615 (9.2) (100)	1565 (9.4) (96.9) ^§^	50 (5.2) (3.1)
Slovenia	1234 (7.0) (100)	1109 (6.7) (89.9) ^§^	125 (13.0) (10.1)
Slovakia	1303 (7.4) (100)	1291 (7.8) (99.1) ^§^	12 (1.2) (0.9)
Total	17,596 (100.0)	13,740 (78.1)	3856 (21.9)

Data were reported as frequency (%) or Mean (±SD) as indicated. ^§^ (%) of native residents in each country.
